# Stratification of diabetic kidney diseases *via* data-independent acquisition proteomics–based analysis of human kidney tissue specimens

**DOI:** 10.3389/fendo.2022.995362

**Published:** 2022-11-17

**Authors:** Qinghua Huang, Xianming Fei, Zhaoxian Zhong, Jieru Zhou, Jianguang Gong, Yuan Chen, Yiwen Li, Xiaohong Wu

**Affiliations:** ^1^ Department of Endocrinology, Geriatric Medicine Center, Zhejiang Provincial People’s Hospital, Affiliated People’s Hospital, Hangzhou Medical College, Hangzhou, Zhejiang, China; ^2^ Key Laboratory of Endocrine Gland Diseases of Zhejiang Province, Hangzhou, Zhejiang, China; ^3^ Laboratory Medicine Center, Department of Clinical Laboratory, Zhejiang Provincial People’s Hospital, Affiliated People’s Hospital, Hangzhou Medical College, Hangzhou, Zhejiang, China; ^4^ Department of Commerce, Westlake Omics (Hangzhou) Biotechnology Co., Ltd., Hangzhou, Zhejiang, China; ^5^ Graduate School, Jinzhou Medical University, Jinzhou, Liaoning, China; ^6^ Laboratory of Kidney Disease, Zhejiang Provincial People’s Hospital, Affiliated People’s Hospital, Hangzhou Medical College, Hangzhou, Zhejiang, China; ^7^ Department of Pathology, Zhejiang Provincial People’s Hospital, Affiliated People’s Hospital, Hangzhou Medical College, Hangzhou, Zhejiang, China

**Keywords:** diabetic kidney disease, progression, autophagy, tissue proteomics, identification

## Abstract

**Aim:**

The aims of this study were to analyze the proteomic differences in renal tissues from patients with diabetes mellitus (DM) and diabetic kidney disease (DKD) and to select sensitive biomarkers for early identification of DKD progression.

**Methods:**

Pressure cycling technology–pulse data-independent acquisition mass spectrometry was employed to investigate protein alterations in 36 formalin-fixed paraffin-embedded specimens. Then, bioinformatics analysis was performed to identify important signaling pathways and key molecules. Finally, the target proteins were validated in 60 blood and 30 urine samples.

**Results:**

A total of 52 up- and 311 down-regulated differential proteins were identified as differing among the advanced DKD samples, early DKD samples, and DM controls (adjusted p<0.05). These differentially expressed proteins were mainly involved in ion transport, apoptosis regulation, and the inflammatory response. UniProt database analysis showed that these proteins were mostly enriched in signaling pathways related to metabolism, apoptosis, and inflammation. NBR1 was significantly up-regulated in both early and advanced DKD, with fold changes (FCs) of 175 and 184, respectively (both p<0.01). In addition, VPS37A and ATG4B were significantly down-regulated with DKD progression, with FCs of 0.140 and 0.088, respectively, in advanced DKD and 0.533 and 0.192, respectively, in early DKD compared with the DM control group (both p<0.01). Bioinformatics analysis showed that NBR1, VPS37A, and ATG4B are closely related to autophagy. We also found that serum levels of the three proteins and urine levels of NBR1 decreased with disease progression. Moreover, there was a significant difference in serum VPS37A and ATG4B levels between patients with early and advanced DKD (both p<0.05). The immunohistochemistry assaay exhibited that the three proteins were expressed in renal tubular cells, and NBR1 was also expressed in the cystic wall of renal glomeruli.

**Conclusion:**

The increase in NBR1 expression and the decrease in ATG4B and VPS37 expression in renal tissue are closely related to inhibition of the autophagy pathway, which may contribute to DKD development or progression. These three proteins may serve as sensitive serum biomarkers for early identification of DKD progression.

## Introduction

Approximately half of all patients with type 2 diabetes will develop diabetic kidney disease (DKD), which is clinically defined as the presence of impaired renal function, elevated urinary albumin excretion, or both (1). DKD is recognized as a leading cause of end-stage renal disease (2). In addition to causing increased mortality, DKD imposes severe health consequences and financial burdens on patients (3). The severity of DKD can be assessed by clinical and pathological methods, and DKD is pathologically graded into four stages (stage I to IV) according to the Renal Pathology Society classification system (4). Both stages 1 and 2 are described as early DKD, and stages 3 and 4 represent progression to advanced DKD (4, 5). In clinical practice, the urinary albumin excretion rate in 24 hours and the albumin-creatinine ratio are commonly used to diagnose DKD and monitor its progression. However, the microalbumin level in the urine is not a sensitive and specific predictor of DKD progression (6, 7). At present, there is a lack of sensitive indicators to predict and identify the progression of diabetic nephropathy, and finding new biomarkers to identify DKD in the early stages is a substantial challenge.

In past decades, proteomic approaches have been used in a number of biomarker studies. High-throughput profiling of the proteome is used to assess biological samples to identify, quantify, and discern the function of all observable proteins in health and disease (8). In the past 10 years, proteomic studies of DKD have enriched our knowledge of the molecular mechanisms involved in the pathogenesis of this condition (9). Using mass spectrometry (MS) techniques, many biomarkers in blood, urine, and tissue have been found that are valuable predictors of and diagnostic or prognostic biomarkers for DKD and its progression (10). Urinary CKD 273 score, serum C3f, MCP-1, transthyretin and cystatin C, and more are powerful predictors for DKD (10–13). However, many biomarker studies are limited by small sample sizes, heterogeneity of results, and a lack of large-scale validation studies. Due to the limitations of detection techniques and the difficulty in obtaining human kidney specimens, previous studies of the pathogenic mechanisms of DKD have been based on blood and urine samples from patients or animal models (14, 15). However, these approaches cannot fully clarify the actual molecular mechanisms of DKD, because they are not based on human renal tissue, which hinders the effort to find new sensitive biomarkers for the early identification of DKD progression.

Recently, the pressure cycling technique (PCT) was developed for use in semi-automatic assessment of small volumes of clinical tissue (16). In addition, Pulse-data-independent acquisition (DIA) technology, which is based on traditional DIA technology, has become available. PulseDIA divides a sample into multiple short gradient injections, each of which has a different mass spectral window, and the mass spectral data collected from the various injections are combined and analyzed to achieve a higher rate of peptide and protein identification than traditional DIA. PCT-PulseDIA is a combination of PCT and PulseDIA that provides higher quantitative accuracy and deeper proteomic coverage and is less time-consuming than traditional methods (17). These features make it suitable for proteomic analysis of kidney tissue from patients with DKD. Thus, in this study we subjected renal biopsy specimens from patients with DKD to proteomics analysis and validated the results by measuring protein levels in blood and urine, with the aim of identifying effective biomarkers for the early diagnosis of DKD and identification of its progression.

## Materials and methods

### Renal tissue preparation

A total of 36 formalin-fixed paraffin-embedded (FFPE) kidney specimens were collected: four from patients with type 2 diabetes mellitus (DM control group), 17 from patients with early DKD (stage IIa-IIb), and 15 from patients with advanced DKD (stage III-IV). There were no significant differences in age, gender, BMI, or blood pressure among the three groups (p>0.05) (**Table 1**). Diagnoses were made by a single pathologist (Dr. JG Gong) according to Tervaert’s pathological classification of diabetic nephropathy. The experiments were carried out with the understanding and written consent of each subject and in accordance with the declaration of Helsinki. The study was approved by the ethics committee of Zhejiang Provincial People’s Hospital.

**Table 1:** Biological characteristics of the patients in different groups.

Grouping	n	Age (year)	Sex (male, %)	BMI (kg/m2)	SBP (mmHg)	DBP (mmHg)
DM controls	4	58.00 ± 11.78	2/4 (50.00)	23.91 ± 4.46	134.00 ± 8.37	81.25 ± 7.32
Early DKD	17	55.53 ± 8.92	13/17 (76.47)	26.34 ± 4.05	142.75 ± 25.00	82.69 ± 15.14
Advanced DKD	15	56.00 ± 12.90	10/15 (66.67)	24.06 ± 2.32	150.67 ± 20.12	77.33 ± 12.92
p-value		0.53	0.61	0.19	0.20	0.40

†Data were presented by mean ± SD (for Age, BMI, SBP, and DBP) or percentage (for Sex). DM, diabetes mellitus; DKD, diabetic kidney disease; BMI: body mass index; SBP, systolic blood pressure; DBP, diastolic blood pressure. P- value was tested by one way ANOVA.

### Pressure circulation technology–based sample preparation

The FFPE tissue samples were prepared for proteomic analysis as described previously (18). Samples were dewaxed, hydrated, and acidified using heptane, a decreasing ethanol series (100%, 90%, and 75%), and 0.1% formic acid in sequence. The samples were next kept under basic hydrolysis conditions in Tris-HCl (100 mM, pH=10) at 95°C for 30 min and then transferred to a solution containing 30 μL lysis buffer (6 M urea, 2 M thiourea), 5 μL Tris (2-carboxyethyl) phosphine (TECP, 10 mM), and 2.5 μL iodoacetamide (IAA) (40 mM). In PCT-Micro Tubes, the samples were lysed, reduced, and hydroxylated at 30°C using PCT (90 cycles, 45,000 psi, 30 s on-time and 10 s off-time). Trypsin (enzyme:substrate ratio, 1:50; Hualishi Scientific, China) and LysC (enzyme:substrate ratio, 1:40; Hualishi Scientific, China) were then added, followed by PCT-assisted digestion (120 cycles, 20,000 psi, 50 s on-time and 10 s off-time). Then, 1% trifluoroacetic acid (TFA) was added to terminate the digestion process. The resulting peptides were desalted with 2% acetonitrile (ACN) and 0.1% TFA and reconstituted. Peptide concentrations were measured with a Nanoscan (Analytic Jena, Germany) at A280, and samples were stored at 4°C for further analysis. All chemical reagents, unless otherwise specified, were obtained from Sigma-Aldrich.

### PulseDIA proteomic analysis

PulseDIA MS was performed as previously described (19). The peptides from each sample were redissolved and analyzed on a nanoElute UHPLC (Bruker Daltonics, Germany) coupled to a timsTOF Pro mass spectrometer (Bruker Daltonics, Germany). Peptide powder was reconstituted in buffer A (0.1% formic acid in water). Peptide digests were separated at a flowrate of 300 nL/min using a 60 min gradient on a 15 cm analytical column with an integrated Toaster column oven at 50°C. The mobile phase B was 0.1% formic acid in ACN. The timsTOF Pro was operated in positive ion data-dependent acquisition Parallel Accumulation Serial Fragmentation (PASEF) mode. The capillary voltage was set to 4500 V. The MS and MS/MS spectra were acquired from 100 to 1,700 m/z and an ion mobility range (1/K0) from 0.7 to 1.3 Vs/cm^2^. The ramp and accumulation time were set to 100 ms to achieve a duty cycle close to 100%. To perform diaPASEF acquisition, we defined two 15 Th isolation windows: from m/z 384 to 1008 and from m/z 475 to 1099. Spectronaut™ (version 14.6) was used to compare all PulseDIA data against a renal-specific spectral library (20) including 539,631 peptide precursors, 448,338 peptides, 13,624 protein groups, and 9205 proteins with a false discovery rate of 0.01. The other parameters were set to the default values.

### Collection and analysis of blood and urine samples

To validate the utility of the selected proteins, we recruited 150 patients with type 2 diabetes: 50 without DKD, 50 with early DKD, and 50 with advanced DKD. Venous blood and spot urine were collected from 20 and 30 patients, respectively, from each group. Sera were separated from the blood samples by centrifugation at 1500 g for 5 minutes. Then, the levels of ATG4B, VPS37A, and NBR1 protein expression were measured by enzyme-linked immunosorbent assay (ELISA) (mlbio Co. Ltd., China) using a microplate reader (BIO-RAD, USA) to read the OD values of the reaction wells to calculate the concentrations. Urine creatinine levels were measured using a biochemical analyzer (AU5800, Beckman-Coulter, USA), and the protein:creatinine ratio was calculated to eliminate the influence of different urine volumes from each patient.

### Immunohistochemistry assay of FFPE

To confirm the expression site of the three proteins in cells of renal tissue, an immunohistochemistry (IHC) assay was used. The detailed methods was as follows: 1) Deparaffinizing and rehydration: Immerse slides in xylene for 10 minutes, and repeat this step one time, then rehydrate two times by sequentially incubating with 100%, 95%, and 75% ethanol for 3 minutes each, finally rinse the slides with distilled water for 1 minute and place them in PBS buffer. 2) Antigen retrieval: Transfer slides to a microwave-proof container and cover with citrate buffer. After heating them in the microwave on medium power for 10 minutes, the slides were cooled in the citrate buffer for approximately 35 minutes. 3) Block endogenous peroxidase: Add appropriate amount of endogenous peroxidase blocker, and incubate at room temperature for 10 minutes;then rinse with PBS buffer for 3 minutes and 3 times. 4) Primary antibody incubation: Primary antibodies for NBR1 (polyclonal), VPS37A (monoclonal), and ATG4B (polyclonal) (proteintech, Wuhan Sanying, China) were diluted at 1:200, and 100 μ L of the antibodies was added for 60 min at 37°C, then the slides were rinsed with PBS buffer for 3 minutes and 3 times. 5) Enzyme-labeled antibody treatment: After adding 100 μ L of enzyme-labeled goat anti-rabbit IgG polymer solution (ZSGB-Bio,China),the slides were incubated at 37°C for 20 minutes and were rinsed with PBS buffer for 3 minutes and 3 times. 6) Color develops: Mix one drop of Liquid DAB plus chromogen immediately with 1 ml of substrate buffer to add on the slides, and incubate them at room temperature for 5 to 8 min. 7) Re-dying: Rinse the slides with tap water, and incubate with hematoxylin staining solution for 20 seconds. Then differentiate and rinse the slides to ensure the color returning to blue. 8) Dehydration and sealing: Immerse slides sequentially into 60%, 80%, 95% and 100% ethanol baths for 5 minutes each, then in xylene for 5 minutes. Repeat this step again in fresh xylene for 5 minutes. Mount the section with sufficient mounting media and cover with a cover slip, then air-dry them in a fume hood. 9) Results reading: The staining results were observed under a light microscope and read on the stained FFPE by a qualified pathologist.

### Bioinformatics and statistical analyses

Statistically significant differences in protein expression in tissue samples, and their concentrations in serum and urine samples from the DM control group and patients with early DKD and advanced DKD, were determined by one-way analysis of variance (ANOVA), and p-values were adjusted using the Benjamini & Hochberg correction. P-values less than 0.05 were considered to be statistically significant. Soft clustering analysis of statistically significant differences in protein expression was performed using the R package “Mfuzz” (21). The average protein expression levels in each group were used as the input data for clustering. The time series were separated according to disease progression, with the initial stage being the DM controls. Metascape analysis was performed to outline the significant canonical pathways (22). The p-value was calculated in Metascape by right-tailed Fisher’s exact test, and p-values less than 0.05 were considered significant.

## Results

### Patient characteristics and study design

**Figure 1:**
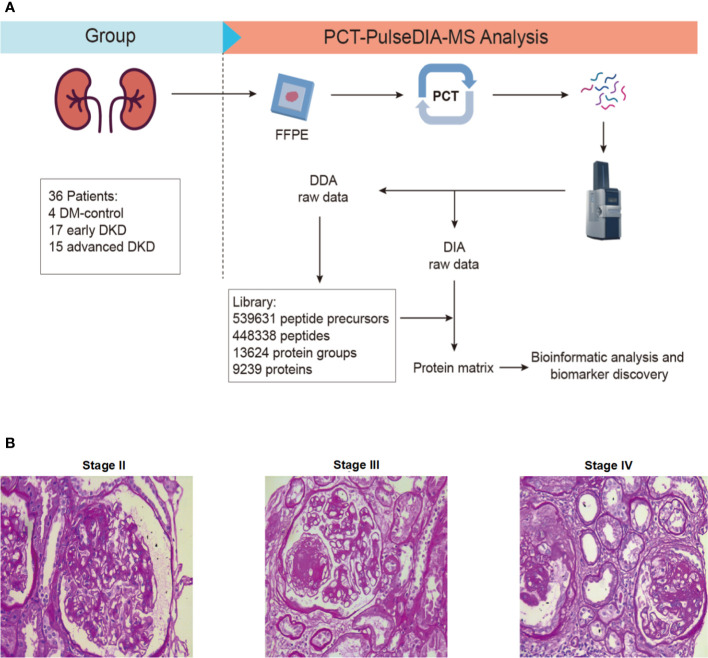
Study design. **(A)** FFPE-PCT-PulseDIA project design and workflow. **(B)** Histopathological characteristics of stage II, III, and IV DKD. FFPE: formalin-fixed paraffin-embedded; DKD: diabetic kidney disease.

In this study, we aimed to identify differentially expressed proteins in renal tissues and confirm them in blood and urine samples from patients with advanced DKD compared with patients with early DKD and DM controls. Advanced DKD and early DKD are defined as stage III/IV and stage IIa/IIb DKD, respectively, diagnosed using the Tervaert criteria for DKD pathological stages. Patients with type 2 diabetes without complications were included as the control group. For the first part of the study, we enrolled 36 patients with type 2 diabetes (T2M), including 4 with DM, 17 with early DKD (stage IIa-IIb), and 15 with advanced DKD (stage III-IV). The FFPE samples were successfully prepared, and the proteins were extracted by PCT. The DDA-MS data were then used to construct a tissue-specific spectral library of the FFPE tissues from the early DKD, advanced DKD, and control patients. All FFPE samples were subjected to PulseDIA to identify differentially expressed proteins. Finally, bioinformatics analysis was performed to determine the regulatory pathways that the differentially expressed proteins participate in. Samples for proteomic analysis were processed *via* a PCT-DIA workflow as described in the Methods section (**Figure 1A**). The histopathological characteristics are shown in **Figure 1B**. The clinical characteristics of the participants are shown in **Table 1**. For the second part of the study, 150 patients with T2D, including 50 DM controls, 50 patients with early DKD, and 50 patients with advanced DKD, were enrolled. There was no significant difference in gender, BMI, or blood pressure among the three groups (p>0.05), although there was a significant difference in age. The detailed clinical characteristics of these patients are not presented here. The expression levels of the proteins selected from the PCT-PulseDIA analysis were detected in 60 blood and 90 urine samples by ELISA to validate their utility as biomarkers.

### Analysis of proteomics profiles

**Figure 2:**
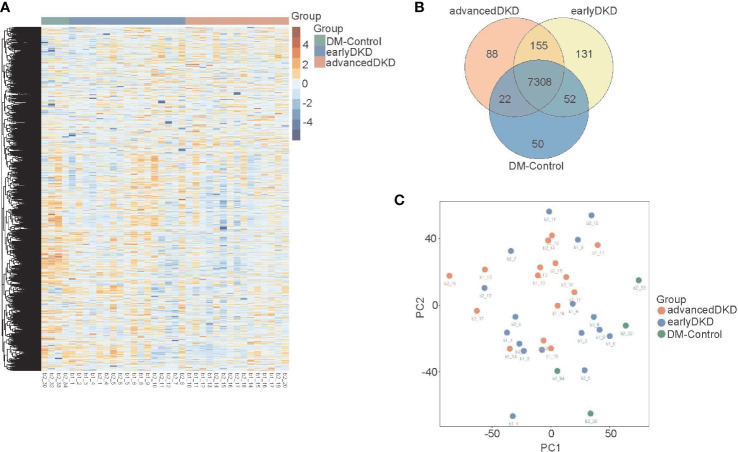
Renal tissue proteome profiles. **(A)** Heatmap showing 9205 protein that were expressed in the renal tissue of patients with DM control, early DKD, or advanced DKD. **(B)** Venn diagram showing overlapping protein expression among the three groups. **(C)** PCA of the 9205 proteins from the three groups. DM: diabetes mellitus; DKD: diabetic kidney disease. PCA: Principal component analysis.

Two technical replicates of each sample in the discovery set were analyzed to enhance the robustness of the proteome maps generated from the FFPE tissues. In total, 36 specimens were analyzed by MS. We identified 9205 differentially expressed proteins in all the samples based on the proteomics data files. These proteins were related to DKD, and their expression levels are shown in the heatmap in **Figure 2A**. One-way ANOVA analysis comparing the three groups showed that the adjusted p-values for 502 of the proteins were less than 0.05. Of these, 52 were up-regulated and 311 were down-regulated with DKD progression, while the changes in expression of the rest of the proteins were irregular. The Venn diagram in **Figure 2B** shows the number of identified proteins displaying significant quantitative similarities and differences among the three groups. There were 8968, 9041, and 7432 proteins identified in the advanced DKD, early DKD, and DM control groups, respectively. A total of 7308 proteins were shared by all three groups, demonstrating that a large set of overlapping proteins (79.4%) was detected, which validated the robustness of the proteome maps to some extent. In addition, 88 and 131 proteins were only identified in advanced DKD and early DKD, respectively, while 50 proteins were only identified in the DM controls. Additionally, principal component analysis (PCA) of the 9205 differentially expressed proteins grouped by pathological stage (**Figure 2C**) revealed that the advanced DKD group shared more proteins with the early DKD group than with the DM control group.

### Biological pathway analysis of proteins differentially expressed in DKD

**Figure 3:**
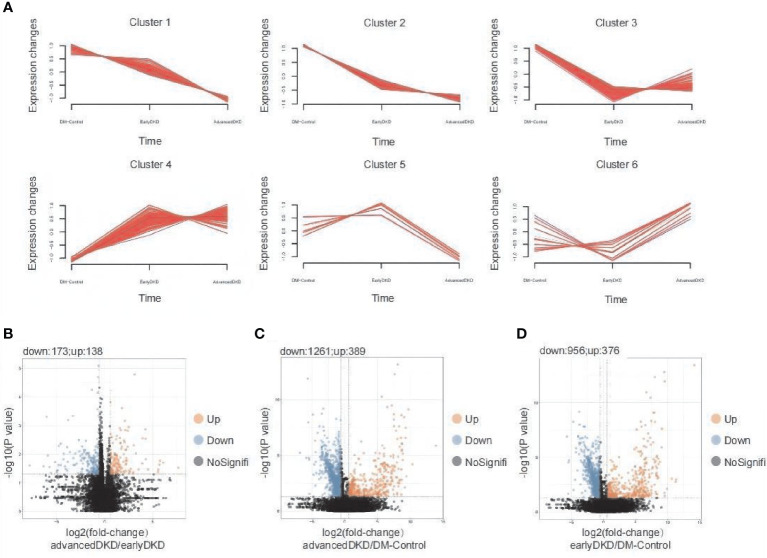
Detection and analysis of differentially expressed proteins. **(A)** Mfuzz analysis of 363 differentially expressed proteins. Volcano plot showing differentially expressed proteins between patients with advanced DKD vs. early DKD **(B)**, patients with advanced DKD vs. DM controls **(C)**, and patients with early DKD vs. DM controls **(D)** using a 1.5-fold–change cutoff and an adjusted p-value threshold of less than 0.05 by ANOVA (p adjusted by Benjamini & Hochberg correction). DM: diabetes mellitus; DKD: diabetic kidney disease; ANOVA: analysis of variance.

The 502 proteins identified by clustering analysis as changing in expression level with disease progression are shown in **Figure 3A**. Each group clustered proteins with different expression trends, namely up-regulation, down-regulation, up-regulation followed by down-regulation, or down-regulation followed by up-regulation. The results from the Mfuzz pathway analysis are shown in **Figure S1**. The proteins that were down-regulated with disease progression are mostly related to metabolism, cellular detoxification, and more, with cellular response to chemical stress and neutrophil degranulation appearing to be the most important pathways. The proteins that were down-regulated and then up-regulated are mainly involved in the regulation of proteolysis, protein phosphorylation, and more, with the main pathways being post-translational protein phosphorylation and neutrophil degranulation. The post-translational protein phosphorylation pathway was also highlighted in the analysis of proteins whose expression was first up-regulated and then down-regulated. In order to better analyze the differences between individual groups, Student’s *t* test was used for pairwise group comparisons (fold change [FC]=1.50 was set as the cutoff value). Compared with the early DKD group, 138 and 173 proteins were up- and down-regulated in the advanced DKD group, respectively (**Figure 3B**); meanwhile, 389 and 376 up-regulated proteins and 1261 and 956 down-regulated proteins were found in the advanced DKD and early DKD groups, respectively, compared with DM-controls group (**Figures 3C, D**). Pathway analysis of the proteins identified in the variance analysis showed that the terminal complement pathway was the most significantly differentially regulated pathway between advanced DKD and early DKD, and that neutrophil degranulation and vesicle-mediated transport may play an important role in DKD development and progression (**Figure S2**).

**Figure 4:**
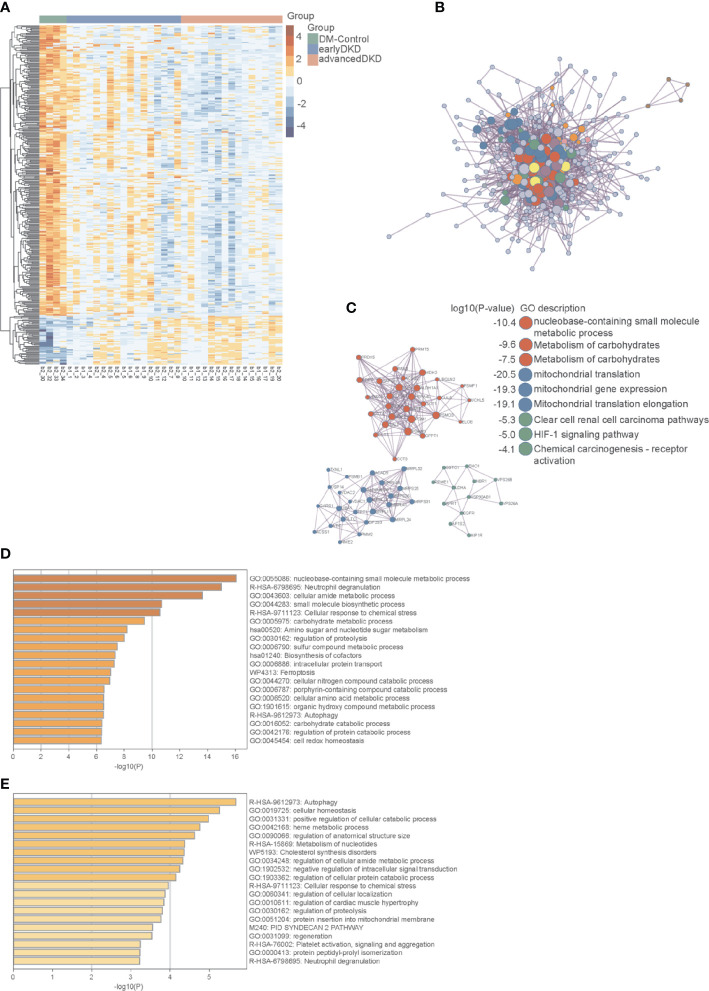
Protein interaction network and pathway enrichment analyses. **(A)** Heatmap showing the expression of 363 differentially expressed proteins in the DM control, early DKD, and advanced DKD groups. **(B)** Network interactions among major differentially expressed proteins. **(C)** MCODE components identified in the protein lists. **(D)** Pathways enriched in differentially expressed proteins using a cutoff value of p<0.05. **(E)** Pathways enriched in differentially expressed proteins using a cutoff value of p<0.01. The p-values were calculated and adjusted by one-way ANOVA followed by Benjamini & Hochberg correction. DM, diabetes mellitus; DKD, diabetic kidney disease; MCODE, Molecular Complex Detection.

Based on ANOVA and Mfuzz analysis with a cutoff value of an adjusted p-value of less than 0.05, 52 proteins showed an upward trend in expression level with DKD progression, while 311 proteins showed a downward trend. When the cutoff value was set to an adjusted p-value of less than 0.01, 17 proteins were up-regulated as DKD progressed, and 100 proteins were down-regulated. The heatmap in **Figure 4A** shows the expression levels of 363 proteins identified as being differentially expressed among the three groups. Then, PCA was performed by Mfuzz analysis (**Figure S3**). These proteins form a network that promotes the occurrence and progression of DKD (**Figures 4B, C**). Next, protein-protein interaction enrichment analysis was carried out using the following databases: STRING (23), BioGrid (24), OmniPath (25), and InWeb_IM (26). Only physical interactions identified by STRING (physical score >0.132) and BioGrid were used in the final analysis. The resulting network contains the subset of proteins that interact physically with at least one other protein on the list. If a protein-protein interaction network contains between 3 and 500 proteins, the Molecular Complex Detection (MCODE) algorithm (27) can be applied to identify densely connected network components. The MCODE networks identified for individual protein lists generated in this study are shown in **Figures 4B, C**. Pathway and process enrichment analysis was applied to each MCODE component independently, and the three best-scoring terms by p-value were retained as the functional description of the corresponding components, as shown in the tables underneath the corresponding network plots in **Figures 4B, C**.

**Figure 5:**
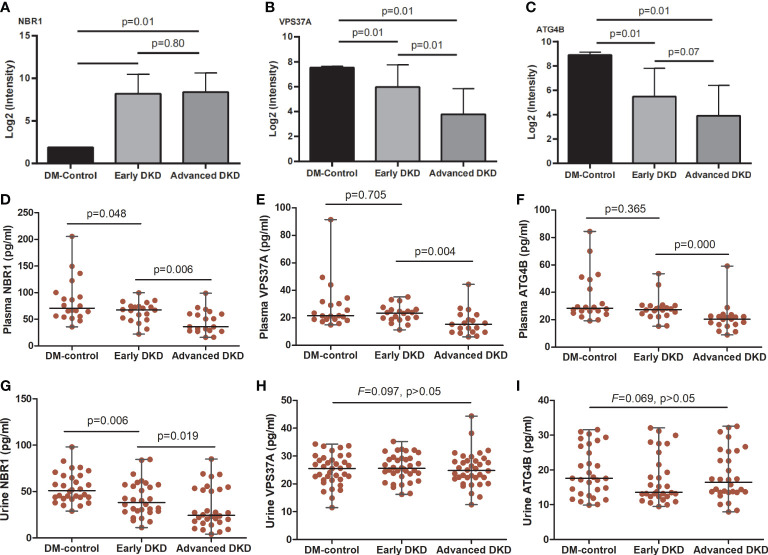
NBR1, VPS37A, and ATG4B expression in renal tissue, blood, and urine. **(A-C)** show the NBR1, VPS37A, and ATG4B expression, respectively, in renal tissue; the p-values were calculated by one-way ANOVA followed by *LSD*-test. **(D-F)** show serum levels and **(G-I)** show urine levels of NBR1, VPS37A, and ATG4B, respectively; the p-values were calculated by one-way ANOVA followed by Mann-Whitney *U*-test. ANOVA, analysis of variance.

The enriched pathways identified by this analysis included multiple metabolic pathways, the iron death pathway, the autophagy pathway, the neutrophil threshing pathway, and other pathways involved in the occurrence and development of nephropathy. Annotation using the Metascape database identified cellular aminosyl metabolism, carbohydrate metabolism, proteolysis regulation, aminosaccharide and nucleotide glucose metabolism, cell REDOX homeostasis, and other biological metabolic processes as playing very important roles in this process, with adjusted p-values of less than 0.05 (**Figure 4D**) and 0.01 (**Figure 4E**). Three proteins in the autophagy pathway, ATG4B (**Figure 5A**), VPS37A (**Figure 5B**), and NBR1 (**Figure 5C**), showed significant changes in expression with progression of the disease. NBR1 was significantly up-regulated in both early and advanced DKD, with FCs of 175 and 184, respectively, compared to the DM control group (both p<0.01). Compared with the DM control group, VPS37A and ATG4B were significantly down-regulated with DKD progression, with FCs of 0.140 and 0.088 in advanced DKD, but 0.533 and 0.192 in early DKD, respectively (both p<0.01).

### ELISA and immunohistochemistry analysis

**Figure 6:**
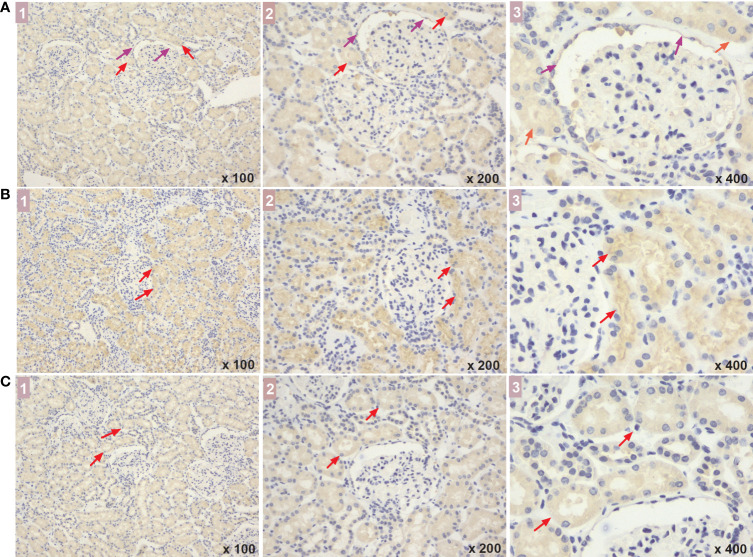
The expressed sites of NBR1, VPS37A, and ATG4B in renal tissue of DM patients. **(A-C)** show the NBR1, VPS37A, and ATG4B expression, respectively, and the marks of 1, 2, and 3 represent the different magnification time under a light microscope (x 100, x 200, and x 400, respectively). What marked with the red arrows means the expressions in renal tubular cells, and the expressions in the cystic wall of renal glomeruli was marked with the purple arrows. DM, diabetes mellitus.

ELISA analysis showed that serum levels of NBR1, VPS37A, and ATG4B decreased with disease progression. There were significant differences in NBR1 expression among the three groups [early DKD (67.67: 22.18-99.60) pg/ml vs. DM control (70.96: 35.73-205.57) pg/ml, vs. advanced DKD (35.94: 16.02-98.78) pg/ml; both p<0.05], and in VPS37A and ATG4B expression between the early and advanced DKD groups [(23.54: 11.29-35.24) and (27.49: 15.31-50.66) pg/ml vs. (15.31: 6.21-44.34) and (20.49: 8.97-59.18) pg/ml, p<0.05; respectively]. However, there was no statistical difference in NBR1 expression between the early and advanced DKD groups, or for VPS37A and ATG4B expression between the DM control and early DKD groups (all p>0.05) (**Figures 5D-F**). Moreover, ELISA analysis of urine samples revealed that NBR1 exhibited remarkable differences in expression among the groups (p<0.05 respectively) (**Figure 5G**), but there were no statistically significant differences in VPS37A and ATG4B expression among the groups (all p>0.05) (**Figures 5H, I**). The FFPEs from the DM-controls were detected by IHC assay, respectively. The results of IHC assaay in each group exhibited that NBR1, VPS37A, and ATG4B were all expressed in the epithelial cells of renal tubule (**Figures 6A-C**), and NBR1 was also expressed in the cystic wall of renal glomeruli (**Figures 6A1-3**).

## Discussion

In the present study, by performing proteomics analysis of human kidney tissue and pathway annotation and enrichment, we found that a total of 363 proteins were differentially expressed among patients with advanced DKD, patients with early DKD, and DM controls, and that these proteins were mainly correlated with ion transport, apoptosis regulation, and the inflammatory response and enriched in signaling pathways related to metabolism, apoptosis, and inflammation. Moreover, we found that the autophagy-related protein NBR1 was significantly up-regulated in early and advanced DKD, but ATG4B and VPS37A were significantly down-regulated with DKD progression; indeed, the autophagy pathway ranked first out of the 20 main pathways that were most highly enriched in differentially regulated proteins. Finally, we found that NBR1, ATG4B, and VPS37A are mainly expressed in renal tubules, and that NBR1 levels in the sera and urine decreased with DKD progression. Our findings indicate that NBR1, ATG4B, and VPS37A may be new, sensitive biomarkers for early identification of DKD development or progression.

DKD can be diagnosed by clinical and pathological methods, and pathological diagnosis is the gold standard (4). However, early identification of DKD and monitoring its progression remain great challenges. In our study, we identified 52 proteins that were up-regulated and 311 proteins that were down-regulated with DKD progression. GO enrichment analysis showed that the differentially expressed proteins were involved in a variety of biological functions, including biological adhesion, biological regulation, developmental processes, localization, growth, immunity, response to stimuli, and prosodic processes, which is consistent with the previous studies of pathogenic factors (28). We also found that proteins that are differentially expressed among advanced DKD, early DKD, and DM are involved in multiple biological functions, including cell redox stability, small molecule biosynthesis, carbohydrates metabolism, and proteolysis regulation, which have also been mentioned in previous studies of the pathogenesis of DKD (29, 30).

Autophagy, which is an intracellular stress response, is currently of great interest to DKD researchers. In a diabetic state, hyperglycemia and hyperlipidemia in the kidney can inhibit autophagy, which leads to reduced autophagic activity in podocytes by blocking the AMP-activated protein kinase signaling pathway and activation of mammalian target of rapamycin pathway (31). Recent studies have found that autophagy dysfunction can worsen renal hypertrophy, tubular damage, inflammation, fibrosis, and albuminuria in diabetic mice, indicating that autophagy plays a protective role in DKD (32). In our study, we found that multiple metabolic pathways, the iron death pathway, the autophagy pathway, the neutrophil threshing pathway, and other pathways are involved in the occurrence and development of nephropathy. The autophagy pathway ranked first out of the top 20 most significantly altered signaling pathways. We identified 12 differentially expressed proteins related to autophagy signaling, and NBR1 expression in particular was significantly increased in patients with DKD. NBR1 is closely related to renal cancer, and also participates in regulation of the autophagy pathway in renal clear cell cancer (33). Our results also showed that there was no obvious difference in NBR1 expression between patients with early and advanced DKD, suggesting that NBR1-mediated dysregulation of the autophagy pathway is likely to play a more important role in DKD development than in DKD progression. We further observed that ATG4B and VPS37A expression decreased significantly in kidney tissues from DM to advanced DKD, indicating the autophagy levels decreased along with DKD occurrence and progression.

In order to validate our results, we measured NBR1, ATG4B, and VPS37A levels in serum and urine samples from DM controls and patients with DKD. We found that the concentration of each of these biomarkers in serum decreased with disease progression. These results were the opposite of what was observed for NBR1 in the renal tissue analysis; further exploration is needed to determine the explanation for this observation. However, the changes in serum ATG4B and VPS37A levels were consistent with those seen in renal tissues, which partially validates the decrease in autophagy during DKD progression and suggests these two proteins could be used as blood biomarkers to evaluate DKD progression. Moreover, while we observed a similar trend in urine NBR1 levels throughout DKD development and progression, urine VPS37A and ATG4B levels did not vary significantly among the groups. Therefore, further prospective studies with a greater number of urine samples may be needed to obtain more definitive results. Moreover, we also used IHC assay to detect the expressed sites of the three proteins in renal cells of DM patients. We found that they were all expressed in the renal tubular epithelial cells, indicating that abnormal autophagy of renal tubule cells may be mainly contribute to the development and progression of DKD. However, NBR1 were also expressed in the cystic wall of renal glomeruli, indicating that its expression may be affected no matter whether the ball or the gym is injured, so it is more likely to show the difference of blood and urine concentration expression in different stages.Therefore, a significant downward trend of this protein was found in the verification test of blood and urine, which further indicated that NBR1 may be an important autophagy protein associated with the development and progression of DKD. Although little is known about the role of ATG4B in DKD, miR-34a is thought to regulate autophagy by targeting ATG4B, leading to acute kidney injury (34). VPS37A is a very important protein in the cellular autophagy pathway, but its role in the pathogenesis of nephropathy is not well characterized. Its correlation with prostate cancer has been explored recently (35). VPS34 interacts with the PI3K complex to promote the nucleation of membrane bubbles and the formation of autophagosomes, and as such, plays a very important role in regulation of the autophagy pathway (36). However, confirming the correlation between VPS34 and nephropathy will require further intensive investigation. Therefore, the key proteins NBR1, ATG4B, and VPS37A, which have only rarely been reported to be associated with DKD in previous studies, are expected to serve as new, sensitive biomarkers for early identification of the development or progression of DKD.

The present study had some limitations. The difficulty of obtaining renal biopsy specimens resulted in a small sample number. The number of blood and urine samples used for the validation experiment was also relatively small. Therefore, the conclusions from our study need to be verified with further experiments in a larger sample size. Despite the size of our study, the results are relatively consistent with previous studies. Moreover, our study identified novel biomarkers associated with autophagy in DKD development or progression, and further confirmed the reliability of proteomics approaches.

## Conclusions

In this study, we effectively identified several candidate markers of DKD progression by proteomic analysis of human kidney tissue. NBR1, ATG4B, and VPS37A, which are all members of the autophagy pathway, are expected to serve as effective biomarkers of DKD development and progression, and suggest that inhibition of autophagy may be a key event in DKD progression. This study may lead to improvement in the early identification of DKD development or progression and help identify new therapeutic targets for DKD.

## Data availability statement

The datasets presented in this study can be found in online repositories. The names of the repository/repositories and accession number(s) can be found in the article/**Supplementary Material**.

## Ethics statement

The studies involving human participants were reviewed and approved by Ethics Committee of Zhejiang Provincial People’s Hospital. The patients/participants provided their written informed consent to participate in this study.

## Author contributions

QHH and XHW designed the project. JGG revised the slides and collected the FFPE samples. ZXZ performed the experiments for discovery set and performed the targeted proteomic sample preparation and analysis. YC performed the Immunohistochemistry and ELISA ananlysis. QHH, XMF, XHW, and JRZ conducted the proteomic data analysis and document search. YWL designed and guided the additional experiments. QHH wrote the manuscript with inputs from all co-authors. XHW supervised the project. All authors contributed to the article and approved the submitted version.

## Funding

This work was supported by the Medicine and Health Science and Technology Project of Zhejiang Province (Grant number 2022KY523), and the Public Welfare Technology Application Research Project of Zhejiang Province, China (Grant number LGD20H070002) and the Research Project of Zhejaing Provincial People’s Hospital (Grant number ZRY2020B014) granted to Huang QH, and the Medicine and Health Science and Technology Project of Zhejiang Province (Grant number 2020KY022 and 2021KY060) to XMF, and the National Natural Science Foundation of China (81970714), Science and technology innovation leading talent project of Zhejiang ten thousand people plan (2021R52022) and Zhejiang Province health innovative talents project to XHW.

## Acknowledgments

We thank Emily Crow, PhD, from Liwen Bianji (Edanz) (www.liwenbianji.cn), for editing the English text of a draft of this manuscript.

## Conflict of interest

ZZ was employed by Westlake Omics (Hangzhou) Biotechnology Co. Ltd.

The remaining authors declare that the research was conducted in the absence of any commercial or financial relationships that could be construed as a potential conflict of interest.

## Publisher’s note

All claims expressed in this article are solely those of the authors and do not necessarily represent those of their affiliated organizations, or those of the publisher, the editors and the reviewers. Any product that may be evaluated in this article, or claim that may be made by its manufacturer, is not guaranteed or endorsed by the publisher.
