# Supplementary figures and images for: Stratification of diabetic kidney diseases via data-independent acquisition proteomics–based analysis of human kidney tissue specimens
Source: Front Endocrinol (Lausanne). 2022 Nov 17;13:995362. doi: 10.3389/fendo.2022.995362 (PMC9714485; doi:10.3389/fendo.2022.995362)

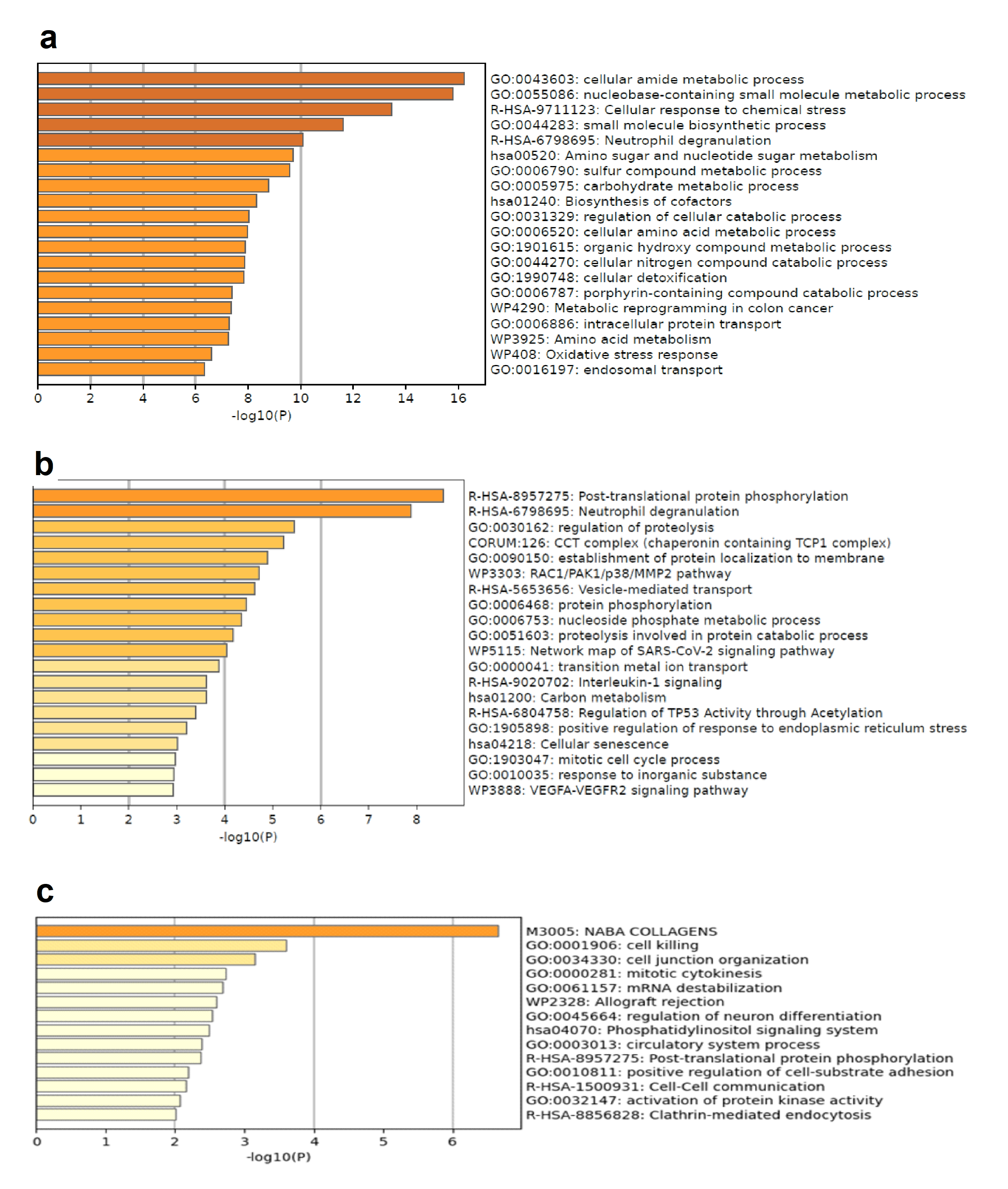

Supplement: Supplementary file 1 [file Image_1.tif]

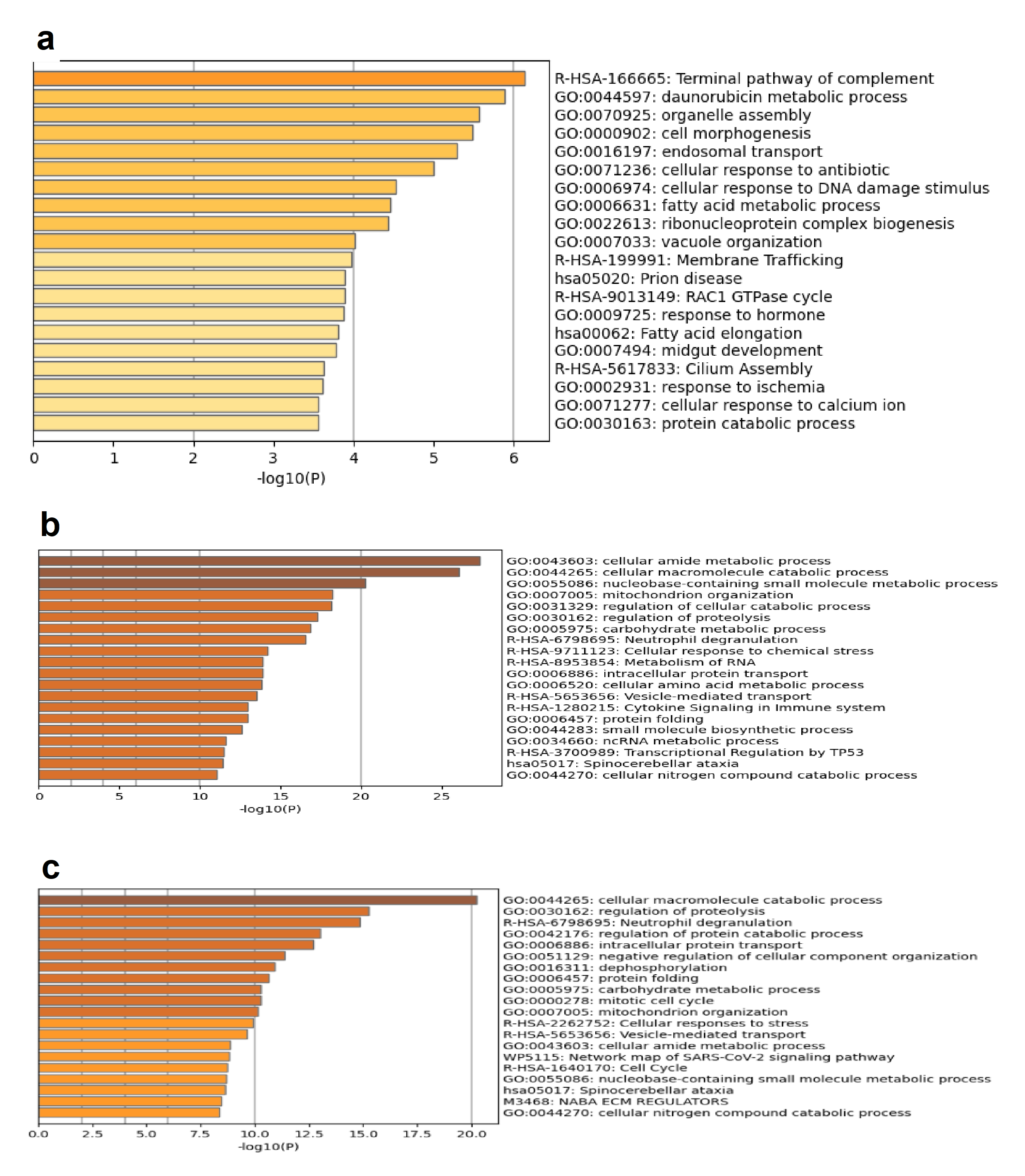

Supplement: Supplementary file 2 [file Image_2.tif]

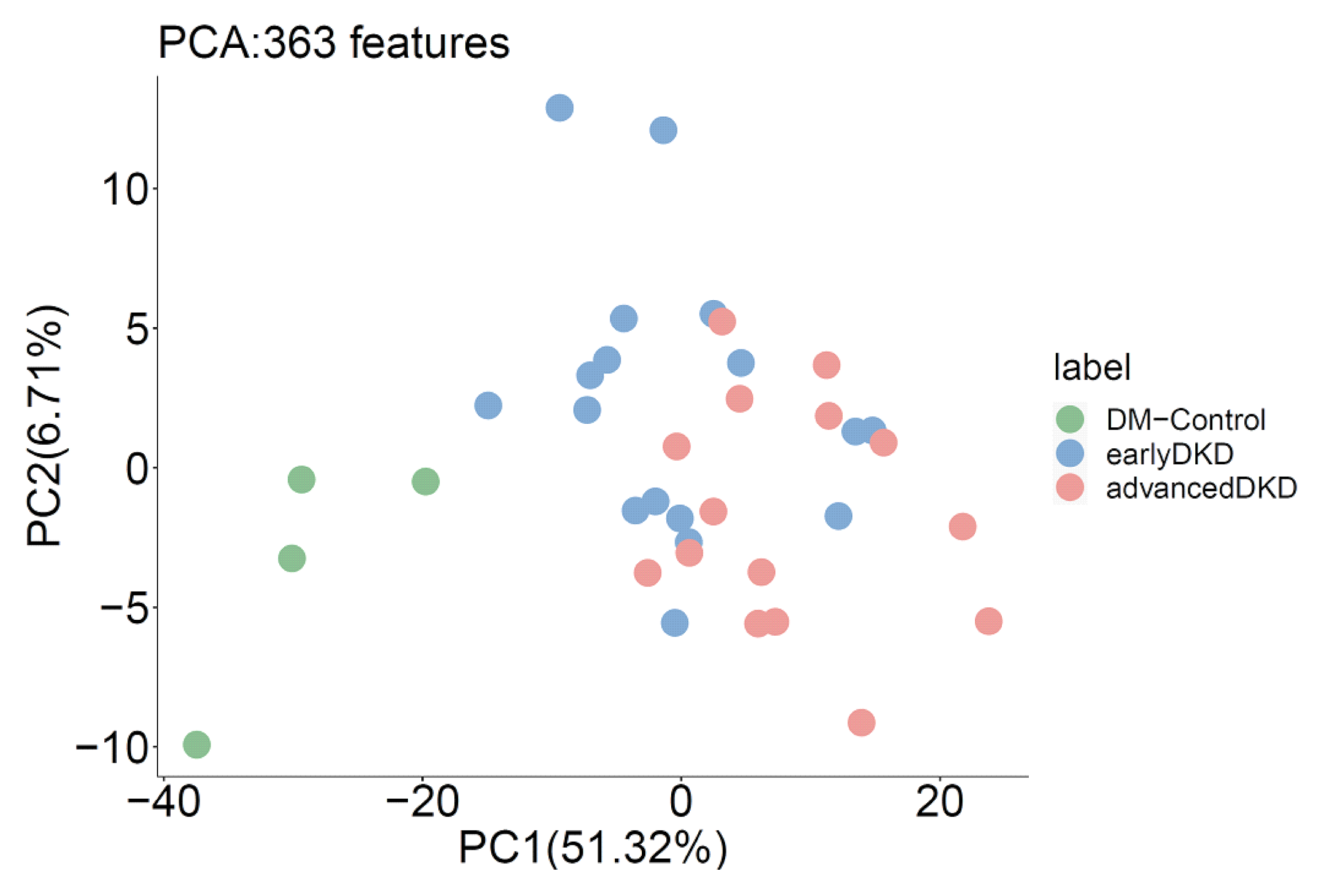

Supplement: Supplementary file 3 [file Image_3.tif]
